# Propagation of tropical squall line-induced storm coastal inundation episodes in Java-Bali, Indonesia

**DOI:** 10.1016/j.heliyon.2023.e19804

**Published:** 2023-09-02

**Authors:** Erma Yulihastin, Ibnu Fathrio, Albertus Sulaiman, Rahaden Bagas Hatmaja, Suaydhi, Haries Satyawardhana, Fadli Nauval, Dwiyoga Nugroho, Thomas Djamaluddin, Widodo Setiyo Pranowo, Rikha Bramawanto, Abdul Basit, Subekti Mujiasih, Mochamad Furqon Azis Ismail, Sopia Lestari, Herlina Ika Ratnawati, Jalu Tejo Nugroho, Danang Eko Nuryanto

**Affiliations:** a Research Center for Climate and Atmosphere, National Research and Innovation Agency (BRIN), Bandung, Indonesia; b Research Center for Oceanography, National Research and Innovation Agency (BRIN), Jakarta, Indonesia; c Research Center for Space, National Research and Innovation Agency (BRIN), Bandung, Indonesia; d Research Center for Remote Sensing, National Research and Innovation Agency (BRIN), Bogor, Indonesia; e Agency for Meteorology Climatology and Geophysics (BMKG), Jakarta, Indonesia

**Keywords:** Squall line, Propagation, Coastal inundation, Thunderstorm, Java, Bali

## Abstract

The short-lived tropical squall lines could trigger weather-related hazards to the northern part of the Indonesia Maritime Continent (IMC), such as Sumatra and Kalimantan. Herein, we investigated the rare propagation event of the long-lived Sumatra squall line associated with a severe storm surge that induced coastal inundation in Java-Bali with devastating impacts from 22 May–2 June 2020. With a comprehensive approach combining observational, numerical, and analytical studies, for the first time, we proposed the possible mechanism related to the long-lived squall line over the IMC, which represents the largest equatorial tropical region with the most complicated air-sea interaction area in the world. Our findings suggest that the long-lived squall line related to the supercell-like thunderstorm initiated from multicell over central Sumatra on May 20, 2020, continuously propagated southeastward until several days later reached Bali. The near-quasi steady convective line has 6 hours of time travel from central Sumatra to west Java. The supercell-like rapidly develops from multicell with a deep convective updraft under the strong and fast cold pool (∼13.8 m s^−1^). The further southeastward propagation of squall line with broken line type seems reinforced by low-level moist transport from the Java Sea. This study also suggested that this unusual event of a long-lived squall line might occur more frequently in the warming upper ocean in the IMC.

## 1 Introduction

As a region with the third longest coastal line in the world, the Indonesia Maritime Continent (IMC) produces the largest tropical rainfall and global latent heating, thus playing a role in controlling the global climate [1]. Consequently, coastal areas of IMC are highly vulnerable due to weather-related disasters that may contribute to the severity of tide flood inundation, which frequently occurs in the lowlands of several cities in IMC [[2], [3], [4]]. However, there still needs to be case studies concerning weather-related phenomena that contributed to the rigorous tide inundation in IMC due to sparse data observation and rare numerical simulation with a high resolution.

From 23 May to June 2, 2020, a coastal surge spread along the north and south of Java-Bali Islands, recorded as the worst inundation due to its devastating impact, which occurred simultaneously and sequentially in several coastal areas from western Java to Bali, Indonesia [5,6]. These extreme episodes result from an astronomical high tide and weather-related mesoscale disturbance that produces storm surges. One weather mesoscale disturbance inducing a rainband of a continuous convective line is a tropical squall line, which consists of cumulonimbus elements and extensive precipitation [7,8].

Previous studies stated that the Sumatra squall line was a common weather disturbance related to severe weather destruction over the Malacca Strait. This squall line type originated over Sumatra and propagated over the Malacca Strait, affecting significantly damaged infrastructure [9,10]. However, the Sumatra squall line rarely propagates southeastward into the Sunda Strait due to its short-lived squall line type with a life cycle, i.e., 30–60 min [7]. This life cycle of a squall line is fundamentally concerned with how typical thunderstorms can be regenerated along a line [11]. The theory proposed a simple way of thinking about how low-level shear enhances regeneration due to a cold pool spreading in an environment without shear. Thus, the circulation at the leading edge is described as a classic density current [12], which should be modified.

**Fig. 1:**
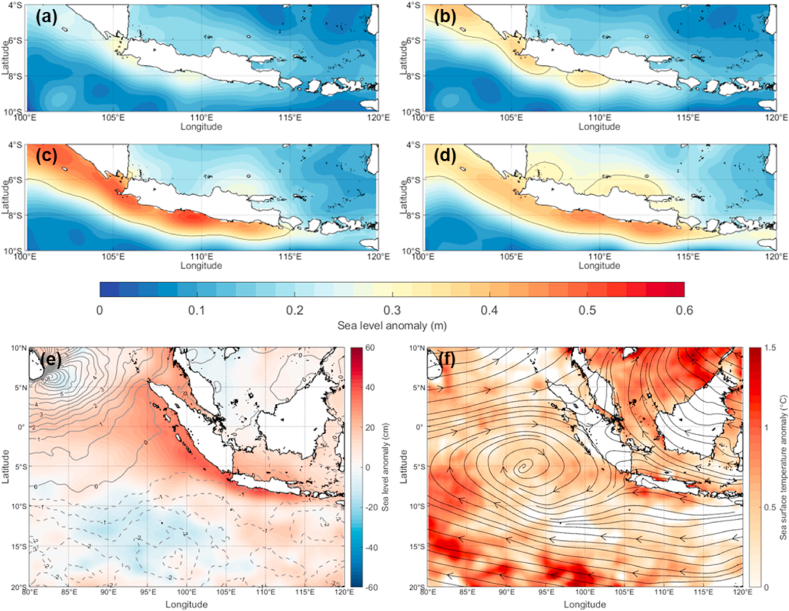
(a–d) Evolution of sea level anomalies from 15 to 18 May, 19–23 May, 24–29 May, 30 May–4 June 2020, respectively. Those periods are denoted as pre-condition, mature, decay, and post-condition of the squall line event. (e) Average sea level and wind stress anomalies from 15 May–4 June 2020. (f) Same as (e), but for average sea surface temperature anomaly and wind divergence derived from the ERA5 reanalysis dataset.

In this study, we considered the long-lived Sumatra squall line during 20–21 May 2020, depicted by the Global Satellite Mapping of Precipitation (GSMaP) (Figure not shown), which induced a long-term period of coastal flood inundation indicated by sea level anomalies of coastal along Sumatra, Java, and Bali Islands, from 23 May to June 2, 2020 (Fig. 1a–c). In this case, since the squall line type is the first record that occurred over the trajectory of the Sumatra-Java regions, we are also concerned about using the possible mathematical approach to describe the squall line propagation. From spatial analysis, the sea level anomalies correspond more to dipole of positive (negative) wind stress anomalies over the northern (southern) Indian Ocean, respectively (Fig. 1d), and a cyclonic vortex (Fig. 1e) than the warming of sea surface temperature (Fig. 1f) over the entire sea in the domain.

We used observational, numerical, and analytical studies to understand how the physical and dynamic processes of squall line propagation triggered the sea level anomalies. In this case study, we used the Weather Research and Forecasting (WRF) model [13] with initial and boundary conditions retrieved from the Global Forecasting System (GFS), whose spatial and temporal resolution is 0.25° and 3 h, respectively, to conduct a high-resolution simulation with convection-permitting of 6 km resolution.

As mentioned in the previous study, ∼5 km grid spacing is sufficient to capture the heavy rainfall event related to the mesoscale process over IMC [14] since we are mainly interested in meso-to-large scale propagation of the squall line in the present study. We further validated the simulation by comparison with the GSMaP satellite and C-band radar observation. The next sections discuss the data, model setup and configuration, the observation and simulation results, and the analytical solutions.

## 2 Data and methods

To investigate coastal flood inundation, we observed sea level anomalies station data from the European Commission Joint Research Center (https://webcritech.jrc.ec.europa.eu/TAD_server/Home?group=IDSL%7C), as well as daily merged satellite altimetry multi-mission data provided by the E.U. Copernicus Marine Environment Monitoring Service (CMEMS, https://marine.copernicus.eu/). The synoptic conditions related to atmosphere parameters over the near-surface using ERA5 data [15] from the European Center for Medium-Range Weather Forecasts (ECMWF), with a spatial resolution of 0.25° × 0.25°, respectively. We also deliberate astronomical parameters affecting the peak sea level over the north coast of Java. Moon and sun ephemeris data were obtained from https://ssd.jpl.nasa.gov/horizons/app.html#/. Furthermore, we examined the GSMaP [16] dataset product and the Agency for Meteorology, Climatology, and Geophysics (BMKG) validated C-band radar [[17], [18], [19]] observation to identify the squall line. Finally, to understand the physical and dynamic processes of the squall-line, we attempted a numerical study using WRF model version 4.2.2 [13] with a single one-way domain (D01) and 6 km spatial resolution. The simulation configuration and scheme are summarized in Table 1. The WRF model configuration follows earlier studies [[20], [21], [22]] that well-simulated the cold pool, propagating convective system, and heavy rainfall compared to satellite observation, respectively.

**Table 1:** Configuration of the Weather and Research Forecasting model for simulating squall line propagation over the western Maritime Continent at horizontal resolutions of 6 km (D01).

Configuration	D01
Number of horizontal grids	850 × 650
Grid spacing (km)	6
Cumulus scheme	Betts–Miller–Janjic scheme
Vertical grid (layers)	45
Microphysics	WRF Single-Moment 6-class scheme
Radiation	RRTMG longwave scheme Dudhia shortwave scheme
Surface layer	Revised MM5 Monin-Obukhov scheme
Land surface	4-Layer Noah LS
Planetary boundary layer	YSU scheme
Initial boundary condition	GFS 0.25 × 0.25

We obtained the initial and lateral boundary conditions from the Global Forecasting System (GFS) [23], initially running at 18:00 UTC (01:00 LT) on May 18, 2020, with 12 h considered a spin-up time for the model. We later validated the simulation of the results with hourly precipitation derived from GSMaP satellite and C-band radar data. The GSMaP dataset product has been applied for the hydrology model to develop flood inundation forecasting in Jakarta and acceptable agreement in simulating inundation in Jakarta [24]. The GSMaP dataset product can also capture the tropical cyclone precipitation, suggesting that the satellite can detect the synoptic disturbance inducing deep convective on a large scale despite under-estimated results comparing ground-based data [25].

### 2.1 Sea level rise and synoptic background observed

**Fig. 2:**
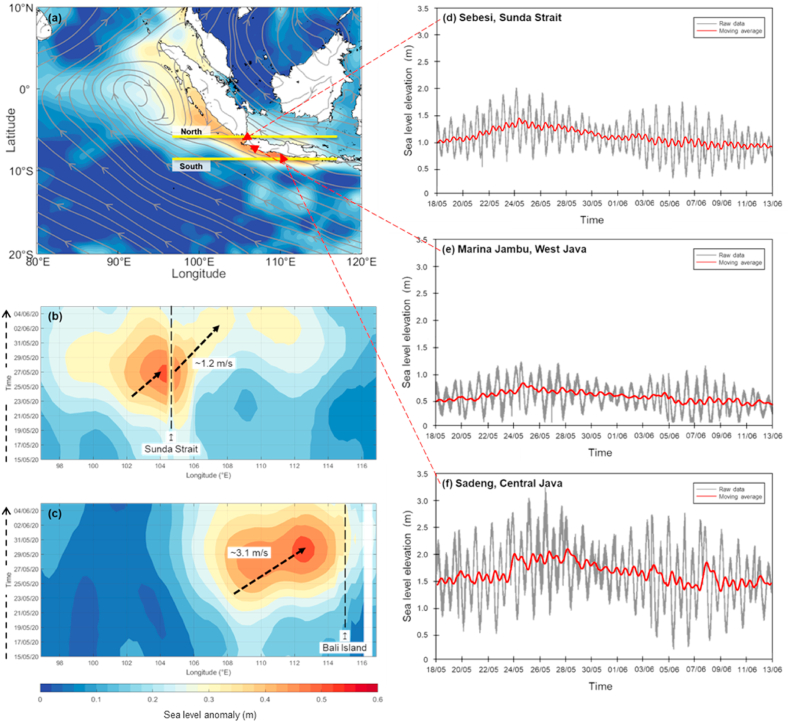
(a) Sea level anomalies and streamline of average surface wind (10 m) from 15 May to June 5, 2020 over the western Maritime Continent derived from ERA5 reanalysis data. (b–c) Hovmöller time-longitude cross section for the north and south coasts of Java (9.6°S). Locations of the north and south of Java refer to the horizontal yellow lines of Fig. 2a. (d–f) The time series of sea level height recorded by Sebesi, Marina Jambu, and Sadeng stations data from 18 May to June 13, 2020, respectively.

The sea level rises over northern and southern Java-Bali, as observed by ERA5 reanalysis data, despite the south increase more than the north part (Fig. 2). Sea level rises over the northern part of Java-Bali initiated from May 22, 2020 in the Indian Ocean (102–105.8°N) (Fig. 2a), fostered intruding to the Java Sea on May 28, 2020 with a continuously prompt increase until June 6, 2020 (Fig. 2b). It is important to note that sea level rise extension from the Indian Ocean to the Java Sea implies that driving forces over the near-surface level at the atmosphere are strongly connected with the surface ocean component. The eastward propagation of Kelvin might also increase the sea level from the Indian Ocean to the Java Sea wave, which resembles prior studies mentioned that its various phase speeds of propagation from 1.3 to 1.7 m s^−1^ [26,27]. The eastward propagation was developed by regular zonal wind strengthening around 20–90 days. In this case study, with a rough calculation, the Kelvin wave propagation is about 1.2 m s^−1^ (Fig. 2b).

However, the rapid speed (3.1 m s^−1^) of eastward propagation was detected in the southern part from 22 May to June 2, 2020, in phase with coastal storm inundation in several low-land cities in Java-Bali (Fig. 2c). This sea level rise propagation was related to a cyclonic vortex, with the core existing precisely over the equatorial line indicated by the streamlined wind at the surface level, which was also strengthened by the Kelvin wave [[28], [29], [30]] and initiated from equatorial zone of Indian Ocean [26,27]. Those sequential sea level rises were confirmed by station data recorded in several locations, such as southern Sumatra, Sunda Strait, and southern Central Java, on 22 May, 22 May, and May 25, 2020, respectively (Fig. 2d–f).

**Fig. 3:**
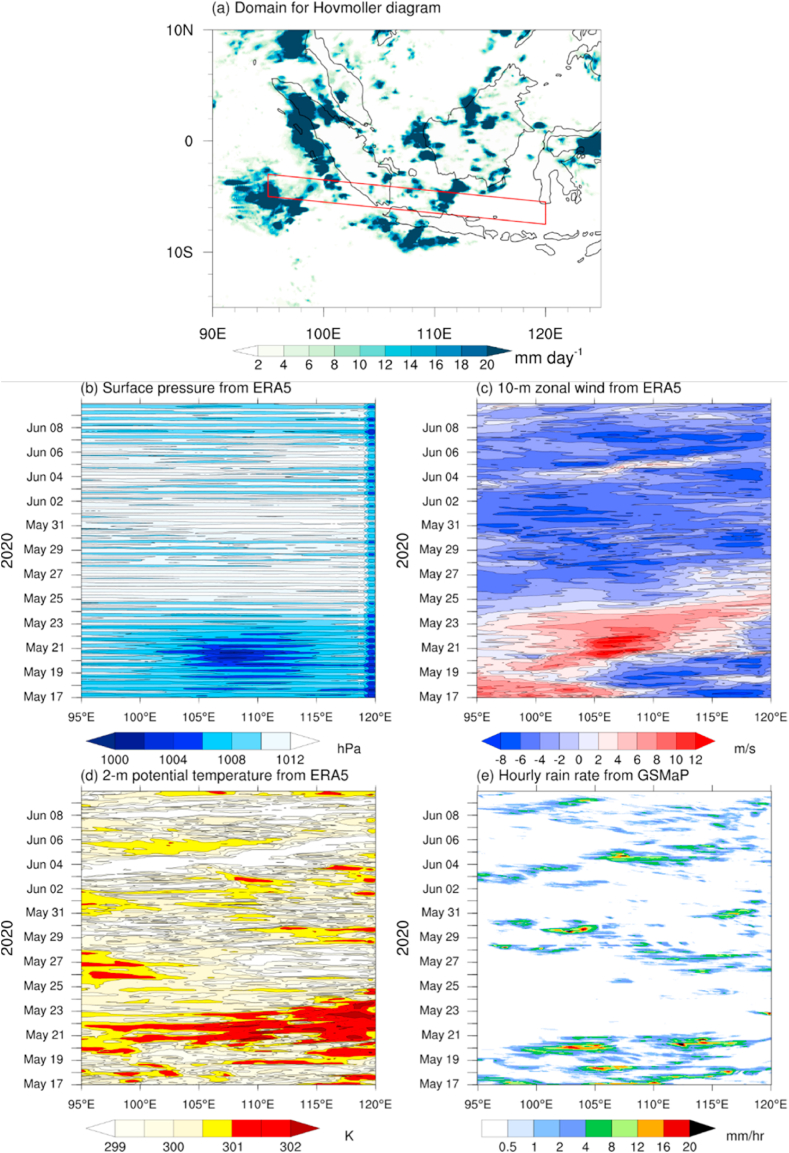
(a) The daily precipitation averaged during 20–21 May 2020 derived from GSMaP satellite data. The red box indicates the Hovmöller diagram domain of a time-longitude averaged cross section during 17 May–9 June 2020 for (b) surface pressure, (c) zonal wind at 10 m, (d) surface potential temperature derived from ERA5 reanalysis data, and (e) hourly precipitation retrieved from GSMaP satellite.

To understand the atmospheric driving force, we create the zonal transect from the Indian Ocean to the Java Sea and explore the near-surface atmosphere parameters, which may lead to the squall line development over the Java Sea depicted by the precipitation average on 20–21 May 2020 (Fig. 3a). From the Hovmöller diagram analysis, the low-pressure system existed over the Indian Ocean and Java Sea (100–115°E), with the lowest pressure concentrated over the Java Sea (105–110°E) during 19–23 May 2020 (Fig. 3b). Therefore, it may conceive a zonal pressure gradient, thus generating a strong eastward from the Indian Ocean (95°E) to the eastern Java Sea (120°E) from 19 to May 25, 2020 (Fig. 3c), followed by the eastward propagation of low-level moisture (Fig. 3d). However, the eastward rainfall propagation intensified significantly on 20–21 May 2020 (Fig. 3e), associated with the squall line propagation that we will further investigate in detail with numerical modeling.

### 2.2 Squall line propagation

**Fig. 4:**
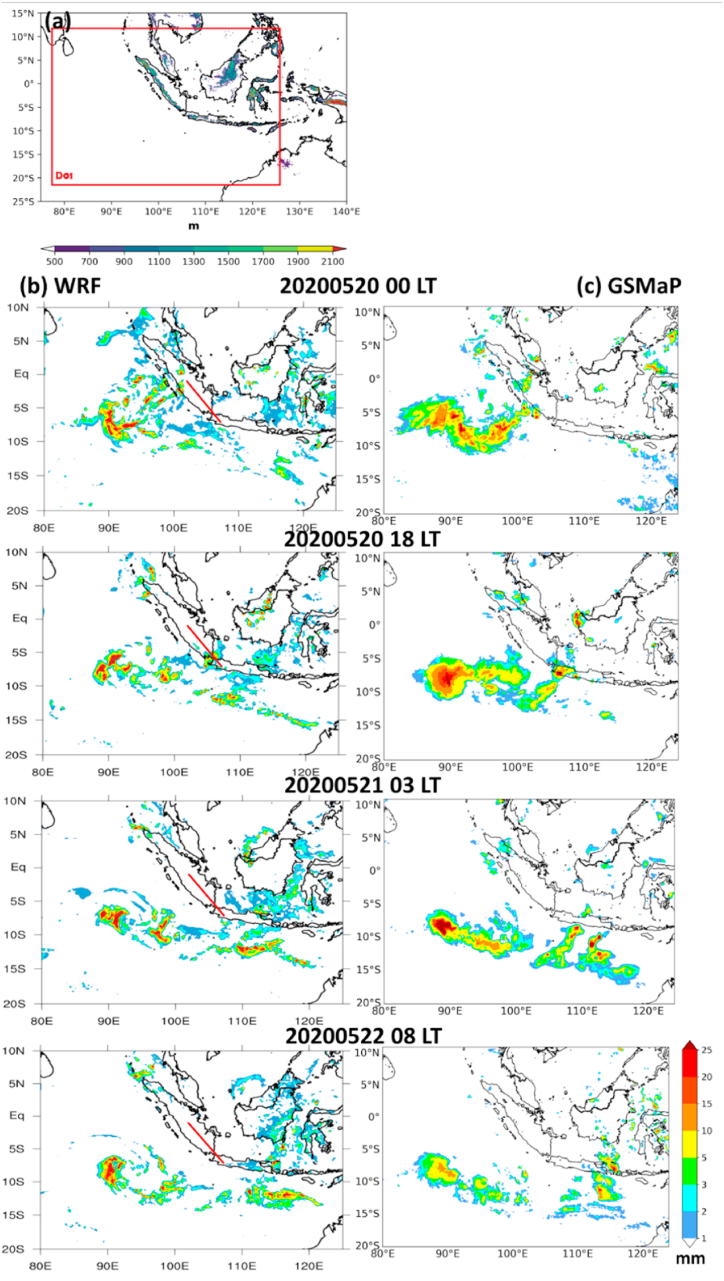
(a) Simulation domain of squall line in WRF model (77–125°E; 22°S–12°N). (b) Spatial map evolution of hourly precipitation simulated from the WRF model from May 20, 2020 at 00:00, 18:00, May 21, 2020 at 03:00 LT, and May 22, 2020 at 08:00 LT, respectively. (c) It is the same as Fig. 4b but for observed precipitation derived from the GSMaP satellite. The solid red lines of each panel indicate the transect used to draw Fig. 6.

Hence, we designed a domain (Fig. 4a) and a configuration (Table 1) of the WRF model to simulate precipitation by considering the existence of a cyclonic vortex and squall line propagation. In this case, the model could capture the propagation of the squall line (Fig. 4b) with the same location compared to satellite observation (Fig. 4c) from initiation over central Sumatra at 08:00 LT on May 20, 2020 to reinforcement over Bali on 08:00 LT on May 22, 2020.

The squall line was mentioned in numerous previous studies that could be initiated from the tropical cyclones (TCs) due to its structure classified into inner and outer rainbands [[31], [32], [33], [34], [35], [36]]. Although the outer rainband of TCs has a 58% similarity degree of prevalence with the squall line [30], several studies also reported that the outer rainband related to TCs could provide squall lines which are indicated by a low-level cold pool [[37], [38], [39], [40], [41], [42]].

**Fig. 5:**
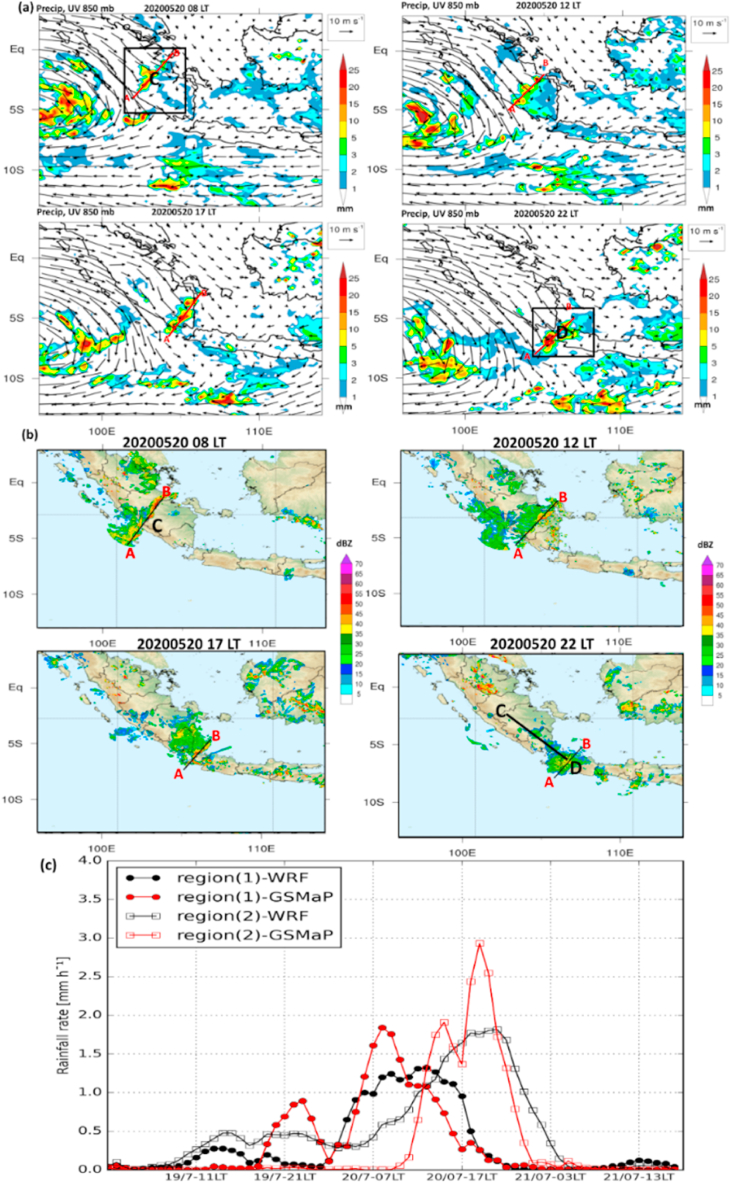
(a) Same as Fig. 4b and c but for simulated precipitation overlaid with the surface wind (10 m) at 08:00, 12:00, 17:00, and 22:00 LT on May 20, 2020, respectively. Two boxes in the north and south regions indicate Regions 1 and 2, respectively, used in Fig. 5c. (b) Same as Fig. 5a but for dBZ derived from C-band radar data. (c) The time series of area average of precipitation between simulated and observed over dark blue solid boxes in Region 1 (5°S–Eq; 102–106°E) and Region 2 (8–3°S; 104–108°E) was marked in Fig. 5a.

Of note, the squall line has strong propagation over the mainland from central to southern Sumatra and enhanced over Sunda Strait as exhibited by simulated precipitation and radar reflectivity, despite the different locations of the maximum convective line captured between model and radar (Fig. 5a and b).

**Fig. 6:**
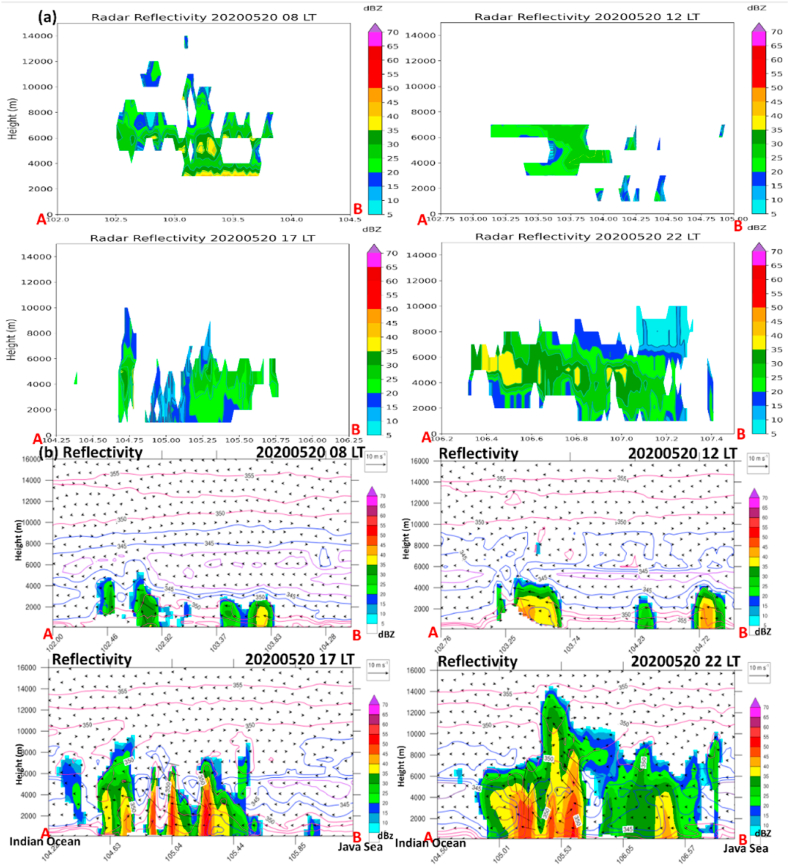
(a) Height-Longitude cross-sections over A-B transect of observed radar reflectivity at different times regarding squall line propagation (see Fig. 5) at 08:00, 12:00, 17:00, and 22:00 LT, respectively. (b) Same as (a) but for simulated radar reflectivity (dBZ; shaded). Simulated potential temperature (θ_e) with 2-K intervals with blue and red contours indicate cold and warm, respectively. The blue color over the surface (<1 km) represents a cold pool. The A-B transect line follows Fig. 4.

However, the intensity and area coverage of maximum precipitation in Region 2 is still comparable between model and satellite observation, which reached the peak at 19:00 LT (∼3 mm h^−1^) and 21:00 LT (∼1.8 mm h^−1^) on May 20, 2020 for precipitation observed and simulated, respectively (Fig. 5c).

In region 1, where the squall line is initiated, precipitation onset occurred at 08:00 LT (∼1.8 mm h^−1^) and 09:00 LT (1.3 mm h^−1^) on May 20, 2020 in GSMaP and model data. However, the model also simulated the following higher peak at 13:00 LT, whereas the observation detected the subsequent lower elevation. Moreover, the model in regions 1 and 2 could appropriately capture the sharp onset of maximum precipitation compared to satellite data observation.

Herein, the squall line fast propagation (08:00–20:00 LT) on May 20, 2020 over the mainland was exhibited over four different locations. Notably, the low-level horizontal wind shear occurred significantly, shown by the formation of a squall line that was created over the leading edge of strong horizontal wind and followed by the weak horizontal wind on the front edge. In this case, the horizontal wind shear related to thunderstorm motion indicated that cold-pool–shear interaction is important in maintaining the structure, strength, and longevity of the squall line [43,37].

### 2.3 The role of the cold pools

Therefore, we analyze the vertical profile of simulated reflectivity for a detailed inspection of squall line characteristics. To validate the vertical structure of the squall line, we used radar observation at four different times and locations following the previous figure (recall Fig. 5).

As a result, it was confirmed by the radar that a squall line was detected through multicell convection in the morning of May 20, 2020 over central Sumatra. However, the discrepancies in vertical structure indicate that the squall line is already in a mature stage observed by radar, whereas the model simulation is still in initiated phase (Fig. 6).

In the mid-day on May 20, 2020, the convective cells propagate southeastward, spread, and tend to decay, as observed by radar, whereas the model simulates the developing stage (Fig. 6a and b).

**Fig. 7:**
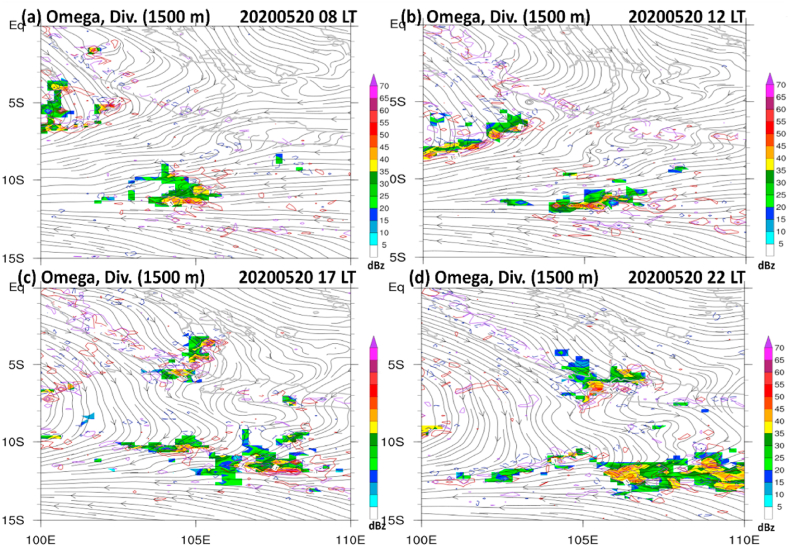
(a–d) Same as Fig. 6, but for simulated radar reflectivity (dBZ; shaded), convergence (red contours at 0.1, 0.5, 1 s^−1^), wind streamline (thin black lines with arrows), and vertical motion (violet contours at 0.1, 0.5 m s^−1^ x 1000) taken at the low-level (1500 m).

However, from the afternoon to midnight on May 20, 2020, the model concedes with the radar that the multicell grow promptly at 17:00 LT and become supercell-like thunderstorms at 22:00 LT on May 20, 2020. It is also interesting to note that the multiplication process of multicell convection occurred in southern Sumatra and Sunda Strait under the back-building mechanism. In the afternoon, strong northerly wind collides from the Java Sea to the south of Sumatra and Sunda Strait, accompanied by low-level (1.5 km) convergences and strong updrafts over a large area in the regions (Fig. 7a–d).

**Fig. 8:**
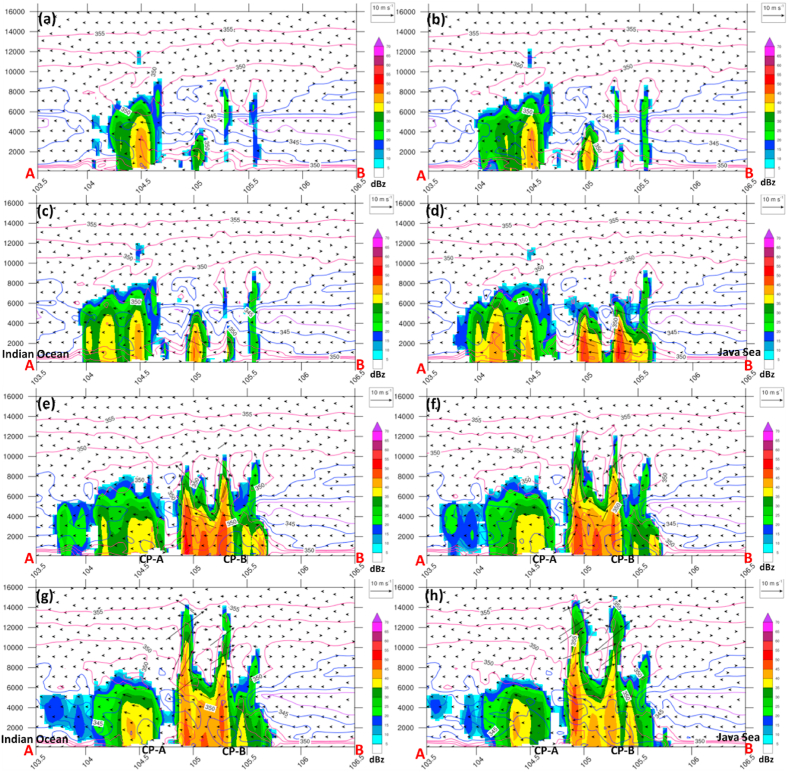
Same as Fig. 7, but for (a–h)16:20, 16:30, 16:40, 16:50 LT, 17:20, 17:30, 17:40, and 17:50 LT, respectively.

Hence, to understand the mechanism of back-building at 17:00 LT on May 20, 2020, we need to analyze previously in a higher resolution (10-min time interval), as shown in Fig. 8. The back building initiated from single to double storms along the 105–105.5°E line (Fig. 8a–d), triggered by strong northeasterly wind and cold pool (so-called A-CP) in the leading edge of the first storm cell over 104.5°E close to A-point (Fig. 8e andf).

Furthermore, the back-building perpetually generates a supercell-like thunderstorm at 22:00 LT on May 20, 2020 with the following stages.

**Fig. 9:**
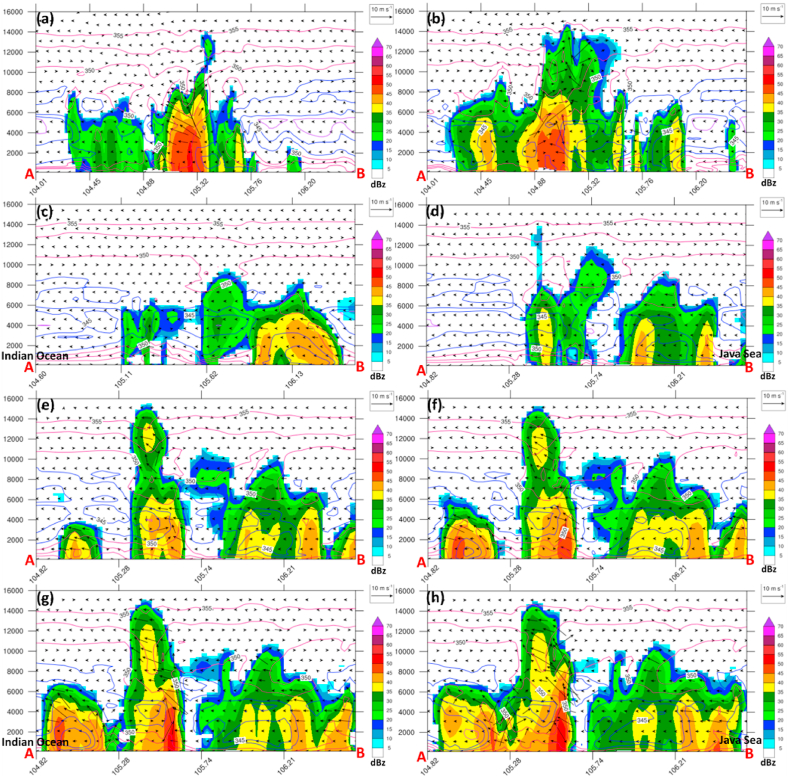
Same as Fig. 8, but for (a–h) 18:00, 19:00, 20:00, 21:00 LT, 21:20, 21:30, 21:40, 21:50 LT, respectively.

- A new cold pool induced the double growth into three storm cells from the northeast (so-called B-CP, which is close to B-point) at 1720 LT (Fig. 8g and h). In addition, a surface cold pool signature was mentioned in numerous studies as a prominent factor in producing new convection cells by enhancing and intensifying the upward motion associated with squall line formation [27,29,30,44].

- The multicell endure develops and merges, becomes a large and strong supercell-like thunderstorm and attains maximum intensity with a strong updraft exceeding 14 km at 18:00–19:00 LT on May 20, 2020 in the first stage (Fig. 9a and b).

The supercell-like decays fast at 20:00 LT on May 20, 2020 over the southwest. Conversely, the new convection cell entrains from the Java Sea to the B line region and rapidly becomes multicell (so-called B-cells) (Fig. 9c and d). At the same time, the new cells also developed southwest close to the A-line (so-called A-cells). Hence, the B-cells also contribute to the entrainment system of A-cells in the mid-level (∼8 km) and have a role in strengthening A-cells to become a supercell-like-like thunderstorm in the second stage at 21:00 LT on May 20, 2020 (Fig. 9e–h).

## 3 Conclusions and discussion

Using observational, numerical, and analytical methods, we have investigated the propagation of a long-lived squall line during 20–21 May 2020 associated with severe storm coastal inundation along Java-Bali from 23 May to June 2, 2020. The simulated results of precipitation and reflectivity have both qualitative and quantitative agreements between satellite and radar data observations. In this case, a long-lived squall line was identified from central Sumatra at 08:00 LT on May 20, 2020 and strongly propagated southeastward over southern Sumatra and Sunda Strait at 17:00 LT on May 20, 2020. The squall line is a quasi-linear convective system with a broken line type, as mentioned in the previous study [38], triggered by the synoptic weather conditions due to a cyclonic vortex existing over the Indian Ocean off the coast of Sumatra.

**Fig. 10:**
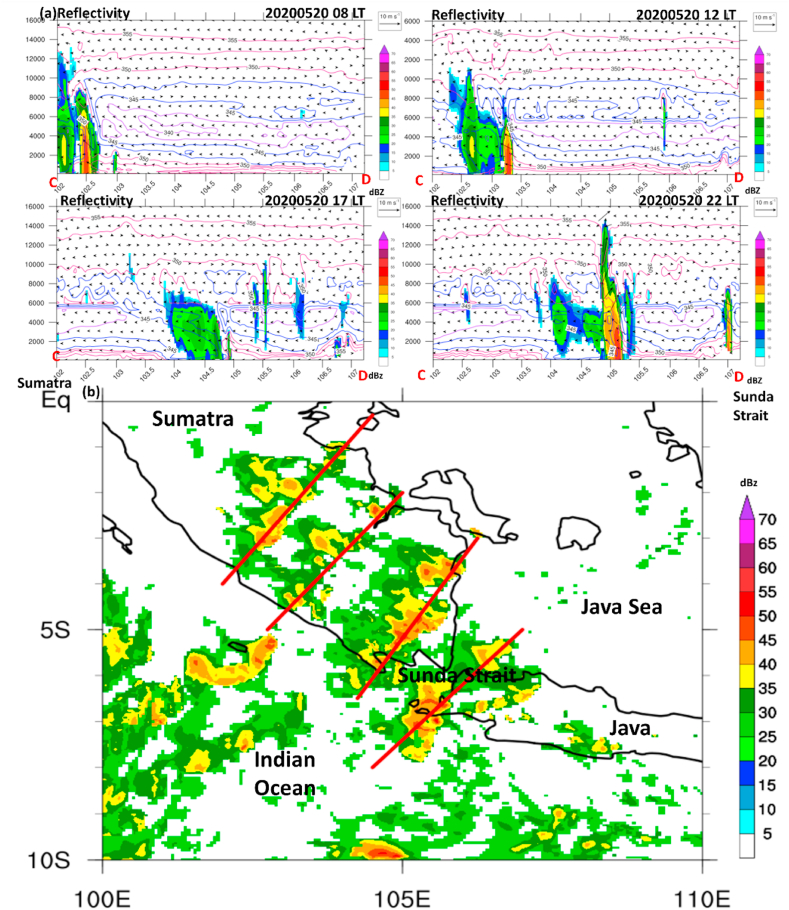
(a) Same as Fig. 9, but for simulated reflectivity over C-D transects drawn in Fig. 5(e). (b) Time travel of squall line propagation in 50-min intervals from northwest to southeast during 20–21 May 2020 for 07:00–07:50, 11:00–11:50, 16:00–16:50, and 21:00–21:50 LT, respectively.

This study suggested the role of the cold pool in multiplying the new convective cells promptly along the A-B line under the back-building mechanism, which supports a dynamics environment for multicell to merge and grow to be a supercell-like thunderstorm revealed by a strong updraft, vertical wind shear, deep convective cloud with height exceeds 14 km (see: Fig. 9). The cold pool also controls the squall line propagation along the C-D line with a rough estimation of phase speed ∼13.8 m s^−1^ (Fig. 10a) and time travel propagation from central Sumatra to western Java around 6 h. However, the location of the squall line indicated by maximum precipitation and reflectivity shows discrepancies between the simulations and radar observations (Fig. 10b).

**Fig. 11:**
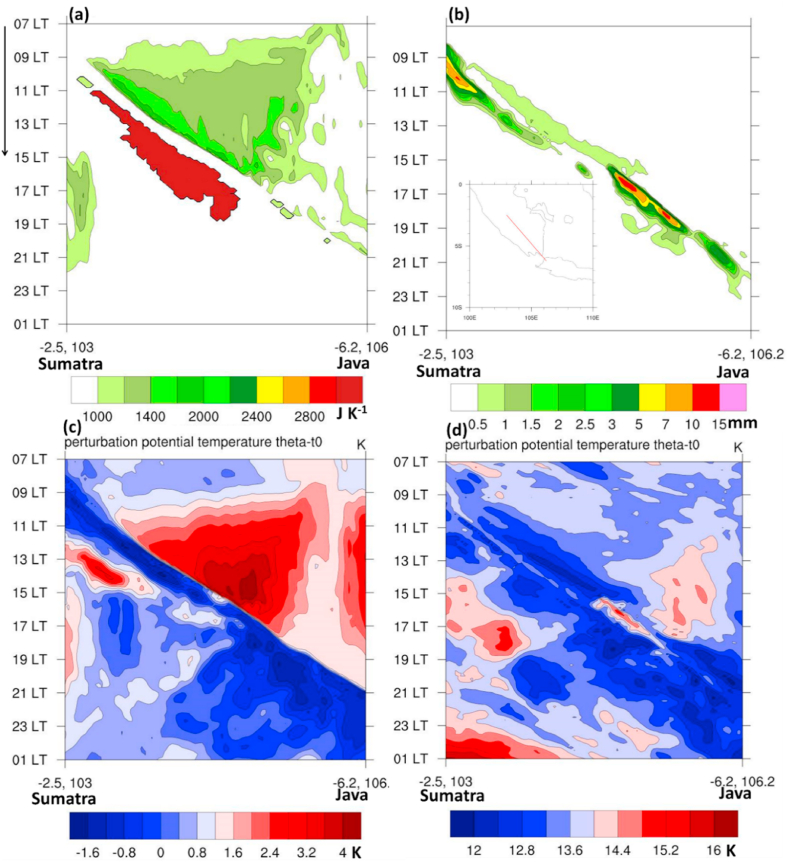
Diurnal cycle on May 20, 2020 through A-B transect for (a) CAPE, (b) rainfall with insert map of transect location, daily mean potential temperature perturbations over (c) 0.5 km, and (d) 3 km. The phase speed of rainfall propagation based on Fig. 10c is ∼ 13.8 m s^−1^.

It should be noted that the role of the cold pool determines the squall line system both in maintaining the multiplication process and in the rapidness of southeastward propagation. The high CAPE and maximum precipitation indicate this fast propagation created by the cold pool (see Fig. 11a and b).

On the other hand, the intensification of the cold pool (0.5 km) was produced by the large discrepancy between the low (high) potential temperatures over Sumatra (Java Sea) (Fig. 11c and d). This mesoscale condition may indicate that the moist transport over the Java Sea has strengthened the squall line propagation, thus decreasing the effect of land surface and terrain in blocking the propagation, as mentioned in previous studies [45,46]. With the near-surface front-like between the dry and moist air, the squall line developed during the self-replication convective system constitutes condensation, evaporation, and sinking [45].

**Fig. 12:**
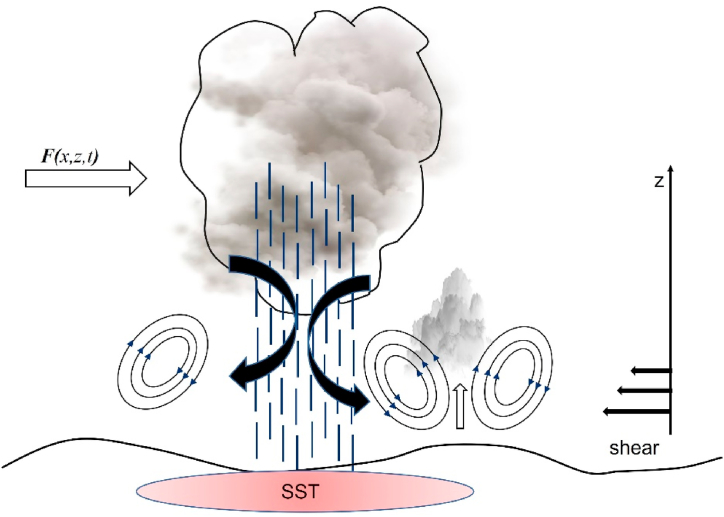
RKW88 squall line mechanism with a warm pool at the sea surface and external forces denoted as RKW88 modified model in this case study. The model suggests that low-level shear interacts with the cold pool circulation and promotes convection, which triggers new cells to form a persistence squall line.

Thus, the long-lived squall line in the real case explained by the modification squall line theory of Rotunno-Klemp-Weisman 1988 (RKW88) has been applied in the previous study [39], considering the role of the cold pool intensity by measuring near-surface wind and cold pool depth. However, in this study, we also propose modifying the RKW88 model by taking into account the external forcing and heating as follows,

$$\psi \left(t,x,z\right)=Ae^{i\left(kx-\omega t\right)}\left\{c_{1}\;\cos\left(\sqrt{x_{1}}z\right)+i{\left(x_{1}-\frac{c_{2}}{c_{1}}\right)}^{-1}\frac{k{\bar{b}}_{0}}{\nu }\left[\frac{1}{x_{1}}{\left(\frac{x_{1}^{2}}{c}\right)}^{\frac{1}{2}}\sin\left(x_{1}z\right)-\frac{1}{x_{2}}{\left(\frac{x_{2}^{2}}{c}\right)}^{\frac{1}{2}}\sin\left(x_{2}z\right)\right]\right\}$$

in this case study, the background wind represents external forcing, and heating refers to warming sea surface temperature, described in detail in Text S1–S2. That RKW88 modified model could be manifested as a possible mechanism depicted in Fig. 12, also demonstrated in an idealized model (Fig. S1) applied in a real case with a propagation speed of 13.8 ms^1^ (Fig. S2).

However, an adequate soliton wave model was still needed to express a more realistic nonlinear effect. In addition, the numerical model result is slower in capturing squall line propagation than observation. Thus, it suggested that the external forcing as a new term based on a soliton solution should be a subject of future study for improving the analytical and numerical models in demonstrating a more realistic mechanism of a squall line over the Indonesia Maritime Continent. This study also suggested that this rare event of a long-lived squall line might be exhibited more frequently in the warming upper ocean in the Indonesia Maritime Continent [47].

## Data availability

The datasets of GFS, GSMaP-MVK, CMEMS, and ERA5 ECMWF, generated during and analyzed in the current study, are publicly available in the permanent archive: DOI: 10.5065/D65D8PWK [23], DOI: 10.1109/TGRS.2007.895337 [16], DOI: https://doi.org/10.48670/moi-00148, and https://doi.org/10.24381/cds.adbb2d47 [15], respectively.

## Funding statement

The 10.13039/501100020638National Research and Innovation Agency (BRIN) was a primary funder this present study under the Widya Nusantara Expedition over Southern Java of Cruise Day Facilitation Program [376/II/FR/3/2022] and Program House of Artificial Intelligence, Big Data, and Computational Technology for Biodiversity and Satellite Imagery, 10.13039/501100020638BRIN [1/III·6/HK/2023]. Widodo Setiyo Pranowo was also partially supported by 10.13039/501100020638BRIN under the Extreme Marine Weather Disaster Program House of Research Organization of Earth and Maritime [B-197/III.4/PR.07.00/1/2023]. The results of this study also support the development of a numerical prediction of atmosphere-ocean knowledge using deep learning artificial intelligent (NAKULA) and maritime information system in Indonesia (SEMAR) at the Research Center for Climate and Atmosphere, 10.13039/501100020638BRIN. The simulations described in this study were completed on the High Performing Computing from the Computational Laboratory of Atmospheric Numerical Prediction (KRESNA) at the Research Center for Climate and Atmosphere, 10.13039/501100020638BRIN.

## Author contribution statement

Erma Yulihastin: Conceived and designed the experiments; Analyzed and interpreted the data; Wrote the paper.Ibnu Fathrio: Conceived and designed the experiments; Performed the experiments. Albertus Sulaiman: Analyzed and interpreted the data; Wrote the paper. Rahaden Bagas Hatmaja; Suaydhi Haries Satyawardhana; Fadli Nauval; Dwiyoga Nugroho; Widodo Setiyo Pranowo; Rikha Bramawanto; Abdul Basit; Subekti Mujiasih; Muhammad Furqon Azis Ismail; Sopia Lestari; Herlina Ika Ratnawati; Jalu Tejo Nugroho; Danang Eko Nuryanto, Thomas Djamaluddin: Contributed reagents, materials, analysis tools or data.

## Declaration of competing interest

The authors declare that there are no conflicts of interest regarding the publication of this paper.
