# Supplementary material for: Propagation of tropical squall line-induced storm coastal inundation episodes in Java-Bali, Indonesia
Source: Heliyon. 2023 Sep 2;9(9):e19804. doi: 10.1016/j.heliyon.2023.e19804 (PMC10559172; doi:10.1016/j.heliyon.2023.e19804)
Supplement: Multimedia component 1 [file mmc1.docx]

Heliyon

**Propagation of Tropical Squall Line Induced Storm Coastal Inundation Episodes in Java-Bali, Indonesia**

Erma Yulihastin^1^*, Ibnu Fathrio^1^, Albertus Sulaiman^1^, Rahaden Bagas Hatmaja^1^, Suaydhi^1^, Haries Satyawardhana^1^, Fadli Nauval^1^, Dwiyoga Nugroho^2^, Thomas Djamaluddin^3^, Widodo Setiyo Pranowo^1^, Rikha Bramawanto^1^, Abdul Basit^1^, Subekti Mujiasih^1^, Mochamad Furqon Azis Ismail^1^, Sopia Lestari^1^, Herlina Ika Ratnawati^1^, Jalu Tejo Nugroho^4^, and Danang Eko Nuryanto^5^

^1^Research Center for Climate and Atmosphere, National Research and Innovation Agency (BRIN), Bandung, Indonesia.

^2^Research Center for Oceanography, National Research and Innovation Agency (BRIN), Jakarta, Indonesia.

^3^Research Center for Space, National Research and Innovation Agency (BRIN), Bandung, Indonesia.

^4^Research Center for Remote Sensing, National Research and Innovation Agency (BRIN), Bogor, Indonesia.

^5^Agency for Meteorology Climatology and Geophysics (BMKG), Jakarta, Indonesia.

Correspondence should be addressed to Erma Yulihastin; erma.yulihastin@brin.go.id

Text S1. Rotuno Theory of Squall Line

When an updraft (convection) occurs, cumulus clouds occur, where after rain falls, followed by a downdraft, the clouds will run out. The formation of two cold poles on either side of the cloud appears at the end of the cumulus cloud formation. A rain cloud that is endless and moves in a line called a squall line. Several observations indicate that squall lines are generally associated with areas of active frontogenesis and most frequently appear in the warm sector of a developing baroclinic wave before the surface of a cold front. Such a system usually occurs in an unstable condition and often brings severe weather. Today we know the squall line as an active thunderstorm in the form narrow band or a storm line that appears in hourly order.

Scientists have made several theories to explain this phenomenon. One possible theory, the squall line, similar to pressure jump lines, can be thought of as disturbances created by acceleration along the cold front, and those travel along the warm sector inversions as gravitational waves [1]. Ley and Peltier develop an analytical model to explain the mechanism of the squall line. They show that the gravity wave emission from a collapsing front can initiate squall line development [2]. The numerical model of the moist convection equation shows that either type of squall line may develop from an initial line-like disturbance depending on the magnitude and orientation of the environmental shear concerning the line [3]. Further, they proposed that when the rainfall, strong shear occurs on the soil surface with negative shear. The negative shear will cause updraft transport causing convections (new cell) which provide input of water vapor so that the cumulus clouds do not dissipate (see Fig. 12 in the main text).

Text S2. Rotuno, Klem dan Weisman (RKW 88) Model

The formulation of the problem is motivated by the phenomenology depicted in Fig. S1, rainfall from a decaying thunderstorm cell chills and loads the air below it. Hence the fluid mechanical problem is to determine the way the cold air spreads at the ground and how this spreading is affected by ambient shear. A simple two-dimensional vorticity-stream function model has been devised to study this problem in a manner consistent with the ideas developed in RKW. The governing equations RKW88 with external forcing (MRKW88) for two-dimensional flow in the *x–z* planes are,

(1)

(2)

(3)

where *η* is the vorticity, *b* is the buoyancy, *ψ* is the stream function which is related to the zonal and vertical velocity as *(u,w)=(∂ψ/∂z,-∂ψ/∂x),* and *ν* is the kinematic viscosity of the atmosphere, and *κ* is the thermal conductivity. Eq. (1), Eq. (2), and Eq. (3) are the nondimensional equation with *L* as the length scale, *H* as the channel depth, *b_0_* as the buoyancy scale, *V_0_=√(b_0_L)* as the velocity scale, and *τ_0_= L/V_0_* is the time scale. The *z* coordinate has the domain *0* to *h*/*H*, and the zonal coordinate length *x=±L_x_/L*. The boundary condition related to Fig. 12 is,

 (4)

where *η_0_=ΔU/Δz* is related to the share effect. If *F(x,z,t)* is an external force and *ϴ(x,z,t)* is sea surface latent heat ignored, then Eq. (1), Eq. (2), and Eq. (3) can be expressed in the term of stream function, yields,

(5)

(6)

(7)

where

(8)

**Linear Case**

We use the Fourier Transform concerning (*t,x*) yields,

(9)

By linearity, we arrive,

(10)

(11)

(12)

Eq. (10) and Eq. (12) gives,

 (13)

where $\bar{b}$ is the solution of,

(14)

Eq. (14) is the harmonic oscillator with the solution,

(15)

We solve Eq. (13) by using the Laplace transform methods. The Laplace transform is an integral problem. A function *f(z)* is integrated with a certain function which in mathematical language is called Kernel, which is expressed in the following form,

(16)

where **$L$** is the symbol of the Laplace transform. The function *e^-sz^* is called the Kernel that transforms a continuous function in variable *z* into the new function in the variable *s*. Applying the Laplace transforms of Eq. (13), we arrive,

(17)

with the boundary condition $\psi(0)=0$, this yield,

(18)

We can re-arrange to be,

...............(19)

We use the table of Laplace transforms to solve Eq. (19). Form the table of Laplace transform as,

 (20)

Due to the Laplace transform is linear, thus we get,

 (21)

where $x_{1,2}= \frac{1}{2}(-\gamma_{1}\pm\sqrt{\gamma_{1}^{2}-4\gamma_{2}^{2}})$.

Further, Eq. (21) can be written as,

(22)

This yields,

(23)

We get,

(24)

which,

Finally,

(25)

By using the table of Laplace transform,

We get the solution of the first term as,

(26)

To solve the second and third terms of Eq. (25), we should find the Laplace transform of *b.*

By using Eq. (26), this yield,

 (27)

Using the Laplace transform for *cos(ωz)*, Eq. (27) yields,

(28)

The inverse Laplace transform of Eq. (28) is given by,

(29)

We have used the pair of Laplace transform,

(30)

The third term of Eq. (25) has the same structure as the second term. Thus it gives the stream function as,

(31)

Thus, the complete solution of RKW88 model as demonstrated in Fig. S1 is,

(32)


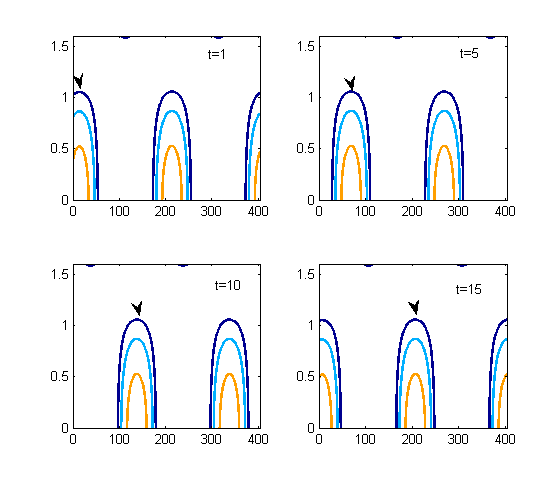


**Figure S1**. Streamline function of the solution on RKW88 model for linear case.

Text S3. Rotuno Theory of Squall Line

An idealized model of a squall line based on the RKW88 solution is depicted in Fig. S2. We choose phase velocity is about 13.8, wavelength 200, x1=1.25, x2=2.35, c1=10. c2=5 and c=5. The solution is plotted in nondimensional units. These results indicate that the analytical solution of the RKW88 model shows the existence of a wave that propagates to the right without damping so that it describes the persistence of the wave (rain). A more realistic model is to account for nonlinear effects where in this paper, we assume that nonlinear solutions are expressed in sech form as soliton solutions [4]. Deriving the soliton equation from the RKW88 model is our future work. It can be seen that the RKW88 model is sufficient to explain the existence of Squall line in this paper (See: Fig. 12 in the main text).


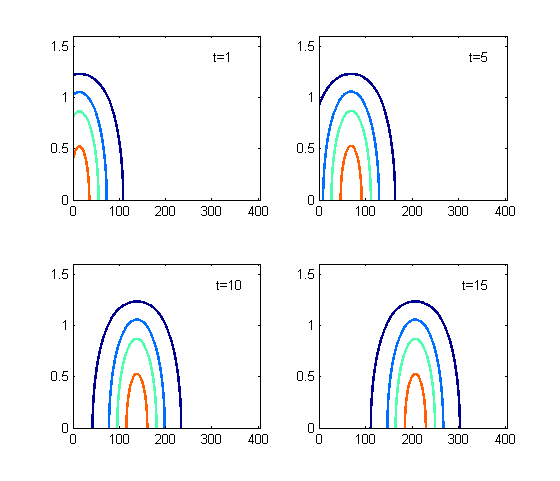


**Figure S2**. Same as Fig. S1 but for nonlinear case. Note that in the nonlinear case we use anzalts that the cos(kx-wt) replaced by sech (kx-wt).

**References:**

[1] Tepper, M., 1950: A Proposed Mechanism of Squall lines: The pressure jump line, *J. of Meteorology*, 7, 21–30.

[2] Ley, B.E., and Peltier, W.R, 1978: Wave Generation and Frontal Collapes, *J. Atmo. Sci*. 35, 1, 3–18.

[3] Rotunno, R., Klemp, J.B., and Weisman, M.L, 1988: A Theory for Strong, Long-Lived Squall Lines*, J. Atmo. Sci*, 45, 3, 463–486.

[4] Arsen'ev, S.A., and Shelkovnikov, N.K., 2010: Soliton Theory of Squall Storm, *Moscow Univ. Phys. Bulletin*, 65, 5, 412–416.
